# Analysis of Factors Affecting Consumers’ Perception of Food Safety Risks in the Prepared Food Market

**DOI:** 10.3390/foods14203463

**Published:** 2025-10-10

**Authors:** Cong Shen, Wenyuan Meng, Xue Chen, Kexin Liu, Xinyao Wu, Qinhe Yu

**Affiliations:** 1 School of Management, Henan University of Technology, Zhengzhou 450001, China; wenyuanmeng0922@163.com (W.M.); 231090500119@stu.haut.edu.cn (X.C.); 2 School of International Education, Henan University of Technology, Zhengzhou 450001, China; 231170300122@stu.haut.edu.cn (K.L.); 231170300326@stu.haut.edu.cn (Q.Y.); 3 School of Management, Jinan University, Guangzhou 510632, China; lcbmarket@126.com

**Keywords:** prepared food safety, perceived risk, influencing factors, bibliometric analysis

## Abstract

The prepared food market has undergone significant growth in response to the contemporary fast-paced lifestyle. This growth has resulted in recurring safety concerns, which have diminished consumer confidence and hindered the industry’s expansion. Analyzing the factors affecting the perceived safety risk of prepared food is essential in this context. This study utilizes consumers of prepared food in Zhengzhou, a newly designated first-tier city in China, as survey participants. This study constructs a research model based on 585 valid questionnaires to systematically investigate the key factors influencing consumers’ perceived risk regarding the safety of prepared food. The findings indicated that perceived risk was adversely affected by the nutritional balance, technical safety, and governance trust. The nutritional balance influences perceived risk indirectly through its impact on technical safety. Governance trust plays a moderating role between technical safety and perceived risk. The higher the governance trust, the stronger the impact of technical safety on reducing perceived risk. This study serves as a valuable resource for governmental oversight and the expansion of prepared food enterprises. Businesses can enhance technical safety, optimize product composition, and cultivate customer trust. To promote sustainable growth in the prepared food industry, the government can improve industry standards and strengthen oversight.

## 1. Introduction

Prepared food, categorized as semi-finished or finished products that have undergone preprocessing, packaging, and preservation via refrigeration or freezing, are well-suited to the modern fast-paced lifestyle and have experienced swift global market growth. Statistical data indicates that by 2025, the global market for prepared meals is projected to attain 600 billion US dollars, while China’s market, the largest for prepared food worldwide, is anticipated to reach 97 billion US dollars. Nonetheless, beneath the thriving growth of the prepared meal business, food safety concerns often emerge. In the 2024 CCTV 3·15 consumer rights protection campaign in China, the inferior quality of prepared preserved pork dishes was revealed, with certain brands identified as utilizing substandard raw ingredients and excessive preservatives. The well-known Chinese restaurant chain brand “Xibei” has also recently been embroiled in a food safety scandal related to prepared food. The public’s concerns about the addition of preservatives in prepared food, the freshness of ingredients, the hygiene conditions during processing, and the rationality of the shelf life, etc., directly affect consumer trust. The limited channels for consumers to obtain food safety information and the information asymmetry further amplify the uncertainty regarding the safety of prepared food. These issues not only become the key factors restricting the expansion of consumption in the prepared food market, but also expose the shortcomings of insufficient response to consumer demands in the current food safety governance of prepared food. These concerns adversely affect consumers’ purchasing behavior, tarnishing the entire reputation of the prepared food business, and result in a notable drop in consumer confidence in prepared food. In this context, comprehensive research on the factors affecting consumers’ perception of food safety risks in prepared food is critically important for protecting consumers’ health rights, fostering the stable growth of the prepared food industry, and enhancing the food safety regulatory framework.

Presently, scholarly investigations into prepared meals predominantly concentrate on consumer behavior and safety technology. Research indicates that consumer acceptance of prepared food is affected by demographic factors, cultural influences, and health values [1,2]. Additionally, studies reveal that consumers prioritize food safety technologies, including processing methods, packaging innovations, microbial risk management, nutritional preservation, and shelf-life extension of prepared meals [3,4]. While prior research has addressed consumer purchase behavior and the acceptance of technical attributes of prepared food, the focus on consumers’ perception of food safety risks associated with these dishes is markedly inadequate [5,6]. Prepared food, as a nascent food category, characterized by industrialized production, intricate supply chains, and multifaceted processing steps, alongside potential hazards such as microbial contamination, additive utilization, and nutrient degradation during storage, transportation, and reprocessing, may engender a heightened risk perception among consumers compared to traditional foods. The food safety of the prepared food market is crucial for the high-quality advancement of the entire industry. Investigating the factors that influence consumers’ perceptions of food safety risks in prepared food can help pinpoint the risk areas that concern consumers, thereby enhancing the food safety quality of the prepared food market and fostering the overall high-quality development of the industry [7,8].

This research aims to investigate consumer risk perception concerning the safety of prepared foods and its affecting elements, specifically within new first-tier cities, based on a review of prior studies. The innovative characteristics of this research mostly reside in two key areas. Firstly, from the perspective of the research topic, current studies predominantly address the consumption behavior or safety technology of prepared food in general, whereas this research specifically examines consumer risk perception concerning the safety of prepared food, thereby addressing a gap in the existing literature and enhancing the body of knowledge related to prepared food research. Secondly, from the perspective of research progress, previous studies on prepared foods have mainly focused on countries or regions with a more developed food industry. This study confines its reach to emerging first-tier cities. Emerging first-tier cities exhibit distinct characteristics in economic advancement, lifestyle tempo, and consuming ideologies, leading to variations in consumer demand and risk perception for prepared meals compared to other cities. Investigations in this area can yield more specific insights for the advancement of the prepared meal market in emerging first-tier cities.

## 2. Literature Review

Upon reviewing contemporary research, we discovered that while previous studies indicate that the safety of prepared food influences consumers’ perceived risk, there is a paucity of systematic investigations into the factors determining consumers’ perceived risk of prepared food. This research systematically investigates the risk variables influencing consumers’ perceptions of prepared food safety, utilizing the CiteSpace bibliometric method and mathematical model creation, in light of the current state of prepared food safety.

In terms of literature selection, to ensure the accuracy and validity of the data retrieval results, the Web of Science, a citation index database, was chosen as the source for retrieval. The data originate from the Social Sciences Citation Index (SSCI) and the Science Citation Index Expanded (SCI-E) databases inside the Web of Science Core Collection. Using the advanced search function of the Web of Science citation index database, the search method was as follows. First, articles with titles containing “prepared food”, “prepared dish”, “pre-made food”, “pre-made dish”, “cooked food”, and “cooked dish” were retrieved, with the search time range set from 1 January 2014 to 10 October 2024 (the search was conducted on 10 October 2024), resulting in a total of 884 articles. Second, the search results were combined using the Boolean operator “OR”, with the language set to English, and the data export format was set to plain text file, i.e., TS = [(“prepared food”, OR “prepared dish”, OR “pre-made food”, OR “pre-made dish”, OR “cooked food”, OR “cooked dish”)]. Finally, the articles were screened through CiteSpace 6.3.R1 software. The parameters were set as TopN = 50, thresholds were all 0, and the time slice was set to 1 year. Using CiteSpace, news reports, editorials, book reviews, and articles with low relevance to the topic or non-research nature were excluded. Ultimately, 659 publications of type “article” and “review” were selected as the basis for the quantitative analysis in this research.

**Figure 1:**
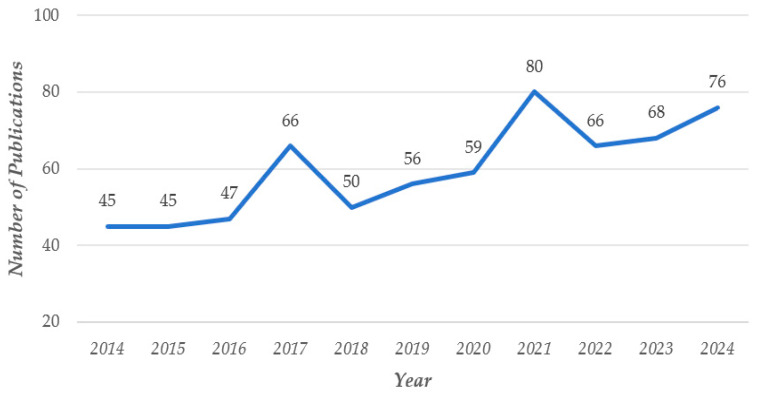
Number of annual publications.

The temporal distribution indicates a general increase in the number of research articles concerning prepared food from 2014 to 2024. After 2019, pertinent research has indicated a swift upward trajectory. The introduction of significant events or development prospects in the prepared food market in 2019 sparked a quick surge in industry interest, resulting in rapid research expansion. By 2022, the proliferation of pertinent research material is increasingly associated with the rising public demand for prepared meals. The market scale is continually expanding, prompting increased attention and research on prepared food inside academics. Figure 1 illustrates the pertinent quantity of articles and their temporal pattern.

**Figure 2:**
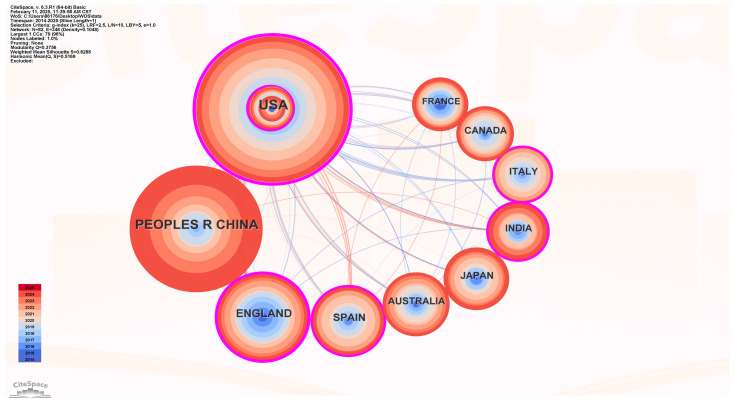
National cooperation network.

Figure 2 illustrates 348 lines and 82 node states corresponding to the analyzed countries. The thickness of the connecting lines represents the degree of collaboration, whilst the size of the circles denotes the quantity of papers published by each country in the research year. Figure 2 illustrates that the majority of study on prepared food has been undertaken in affluent nations, including the United States and Europe. The centrality metric can be utilized to assess the degree of collaboration. The leading three nations on the intensity of international cooperation were the United States (0.41), the United Kingdom (0.37), and Spain (0.19). The United States released 143 articles, securing the top position globally. China and the United Kingdom produced 113 and 53 papers, respectively. Subsequently, Spain and Australia were included. Despite the evident global market presence of China, as indicated by the number of nations reported, there remains a deficiency in pertinent internet research regarding the food safety regulations of prepared food in this context. The quantity of research papers examining the safety regulation of internet prepared meals is more in China compared to the UK, Spain, Australia, Japan, India, Canada, Italy, and France. However, the quantity of research about prepared food in China and the United States remains markedly disparate.

**Figure 3:**
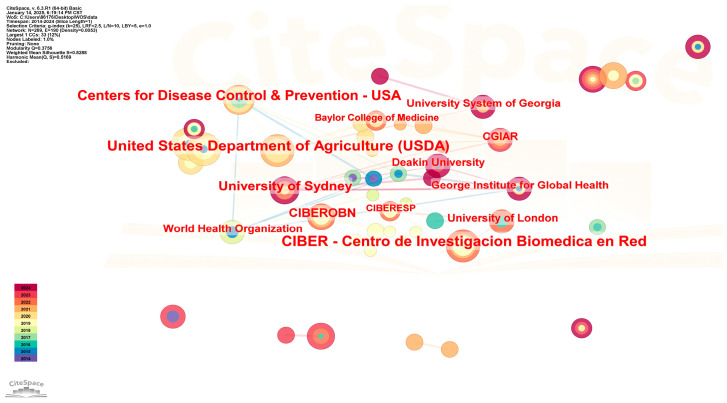
Institutional cooperation network.

Figure 3 illustrates the institutional collaboration network for research effect with prepared food. The three most productive scientific institutions are Jiangnan University in China (16 publications), CIBER-Red Biological Research Center in Spain (8 publications), and Bloomberg School of Public Health in the United States (8 publications). The collaborative network of research institutions depicted in Figure 3 indicates that the investigation into the safety regulation of online prepared food remains in its nascent phase, as demonstrated by Jiangnan University, which, despite having the highest publication count, possesses only 16 pertinent studies in this domain. An examination of the pertinent collaboration network metrics (N = 269, E = 190, density = 0.0053) indicates that the connections among various research institutions require enhancement.

Table 1 indicates that the leading five journals with the highest number of published studies on the regulatory implications of prepared food are Plos One (176) and Food Chemistry (160). American Journal of Clinical Nutrition (150), Food Control (134), and Journal of Agricultural and Food Chemistry (131). These journals hold a significant role in the discipline. The most often cited writers in this domain are Silvennoinen, Kirsi (413), and Adams, Jean (275). This signifies that these authors have made substantial contributions in pertinent scientific domains.

**Figure 4:**
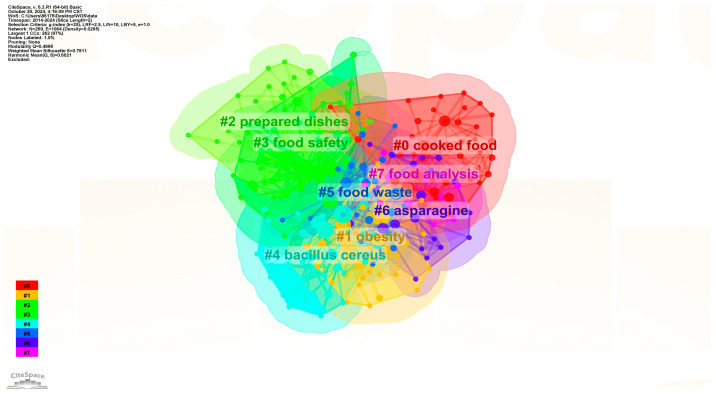
Literature cluster analysis.

The cluster analysis thoroughly encapsulated the prevailing research hotspots internationally and selected literature classified as “Cited Reference”, with a Top N limit of 30 for an exhaustive study. The findings indicated that the eight clustering hotspots were cooked food, obesity, prepared food, food safety, Bacillus cereus, food waste, asparagine, and food analysis. Figure 4 illustrates that the clustering network has N = 269 nodes, E = 1064 edges, and exhibits a density of 0.0295. The modularity index Q value is 0.4898, significantly exceeding the threshold of 0.3, signifying a high degree of modularity in the network structure. The Mean Silhouette coefficient of 0.7811 significantly exceeds the benchmark of 0.4, further confirming the efficacy of the cluster map.

We clearly designate the eight main research hotspots as clusters #0 to #7 by cluster analysis. Eight primary study focal points are distinctly recognized and may be categorized into three fundamental domains: health impacts, food safety and ingredient analysis, and the advancement of the prepared food sector. Cluster #1 (obesity) and Cluster #7 (Asparagin) examine the health implications of prepared food, particularly its significant correlation with obesity. Research indicates that the elevated levels of salt and fat, coupled with the low nutritional value of prepared food, may be significantly associated with the rising prevalence of obesity. This research offers a crucial scientific foundation for the development of public health policy. Cluster #2 (prepared food) and Cluster #3 (food safety) of food safety and composition studies concentrate on the production process, quality control, and compositional analysis of prepared food, highlighting food safety concerns and potential dangers of microbial contamination, including Bacillus cereus. These investigations have propelled the advancement of industry regulation and technological innovation, underscoring the issues faced by the prepared food sector in guaranteeing food safety. Clusters #4 (Bacillus cereus), #5 (food waste), and #6 (cooked food) pertain to issues of food quality deterioration and waste that may arise during the storage and transportation of prepared meals, and suggest strategies to mitigate waste and enhance food preservation quality through technical methods. This cluster analysis has thoroughly examined the diverse subfields within prepared meals, offering significant insights for future research trajectories. These studies elucidate the issues confronting the prepared food sector and establish a foundation for the formulation of pertinent legislation, enhancement of industry standards, and future innovation.

**Figure 5:**
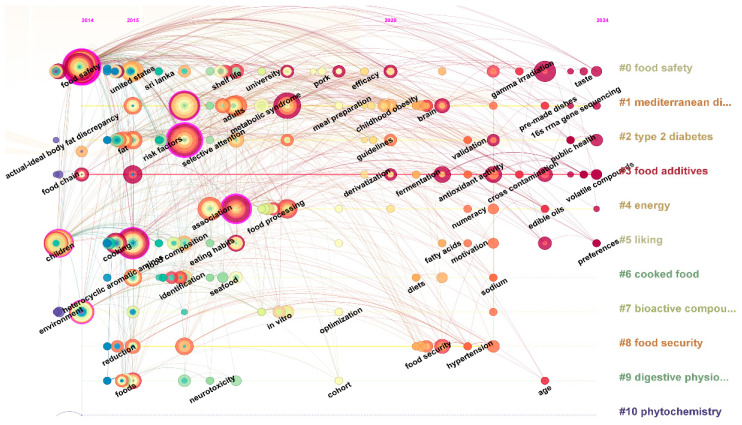
Keyword timeline analysis.

We created a keyword timeline analysis chart reflecting the research advancements and trends across different countries, based on the literature review. Figure 5 illustrates that the investigation into the food safety of prepared food can be categorized into three distinct stages.

The initial phase (2014–2015): the foundational development and market reaction to prepared meals. The keyword timeline analysis chart indicates that the predominant terms in the initial phase encompass food safety, actual optimum body fat difference, obesity, environment, preparation, and food chain. During this preliminary phase, the research concentrated on novel dining styles, the initial development of prepared food, and the establishment of their supply chain. Researchers have extensively examined the disparities in body fat, food production and supply chain optimization, poultry processing technologies, and the influence of environmental factors on the food safety regulation of prepared food. These studies offer policymakers helpful recommendations for reasonable regulation, enhancing efficiency, and protecting food safety.

The second stage (2015–2020): prepared meals and wellness from an ecological standpoint. The keyword timeline analysis chart indicates that the predominant high-frequency terms in the second stage are risk factors, cookery, association, metabolic syndrome, and adults. In contrast to the initial phase, the second phase of research commenced an examination of prepared cookery, food safety, and human health as an interconnected ecosystem [9]. The research emphasis has transitioned to risk identification and the safeguarding of human health inside the system. Researchers aim to mitigate food safety hazards and diminish potential risks to human bodily and mental well-being by examining the culinary techniques of prepared meals and the production and lifestyle of contemporary humans [10].

The third stage (2020–2024): Collaborative governance and behavioral decision-making. The collinear keyword time zone map indicates that high-frequency terms in the third stage encompass radiation exposure, behavior, activity, pattern, and lipid oxidation. This research era concentrated on the challenges of supervising food safety in prepared meals and the mechanisms of collaborative governance, while thoroughly examining the behavioral decision-making of system players, strategic choices, and the creation of government policies. The researchers examined regional studies across various situations to offer a more thorough and nuanced perspective on the supervision of food safety for prepared items. Future research will further examine food safety and regulatory governance as an integrated system, focusing on its sustainability and perceived selection capacities. Concurrently, scholars will focus more on the safety and regulatory control of prepared food in regional growth, particularly in first-tier cities or regions where the cuisines originate. These developments suggest that research on prepared food and associated topics will become more thorough and extensive, offering robust support for policy creation and industrial advancement.

**Figure 6:**
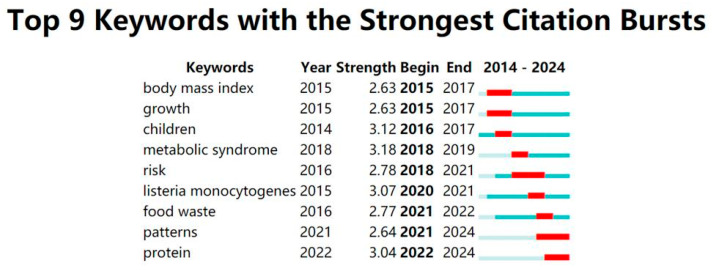
Top 9 most cited keywords.

Figure 6 illustrates that, according to academic research data from 2014 to 2024, several terms with notable citation surges developed, including “Listeria monocytogenes”, “protein”, and “food waste”. These keywords not only underscore the principal study domains of academia but also offer significant insights for prospective research trajectories.

The term “Listeria monocytogenes” underwent a citation surge in 2020, attaining a peak intensity of 3.07 and persisting into 2021. This outbreak may be associated with heightened global apprehension around food safety and foodborne illnesses, especially Listeria, a foodborne organism that presents a significant public health threat globally. Listeria, capable of proliferating at low temperatures and prevalent in various food items, poses a continual concern; thus, researchers have dedicated significant effort to devising effective detection and control measures.

The term “Protein” experienced a citation surge in 2022, reaching an intensity of 3.04, and this trend persisted throughout 2024. This trend signifies the increasing significance of protein research in disciplines such as biology, nutrition, and food science. Proteins are essential elements of biological processes, playing crucial roles in cellular architecture, metabolism, and immune response. Investigating the structure and function of proteins, along with their involvement in diverse disorders, is essential for the advancement of novel therapeutics and the enhancement of human health. Recent advancements in proteomics and bioinformatics have significantly enhanced our comprehension of protein networks and their involvement in disease mechanisms.

The term “food waste” witnessed a citation surge in 2021, achieving a strength of 2.77, and persisted into 2022. This trend underscores the worldwide emphasis on food waste and the increasing scholarly interest in sustainability and resource management. Food waste constitutes a significant environmental challenge and profoundly affects global food security and resource efficiency. Approximately one-third of global food is squandered, which not only contributes to greenhouse gas emissions and wasteful resource utilization but also intensifies food insecurity. Consequently, minimizing food waste has emerged as a priority in policy and scholarly research aimed at optimizing food systems, enhancing food storage technology, and fostering sustainable consumption practices. The surge in citations for these terms has yielded significant insights into major research issues over the past decade and offered critical guidance for future research trajectories. As global health concerns, food safety, and sustainable development challenges escalate, research in these domains will increasingly gain significance, potentially resulting in crucial scientific breakthroughs and technology advancements.

An examination of pertinent research on prepared food reveals that contemporary studies predominantly concentrate on the following topics. Initially, from a supply chain viewpoint, it examines the identification of safety issues in prepared food. Secondly, from a regulatory standpoint, it addresses the establishment of the food safety standard system for prepared food. Thirdly, from a technical standpoint, it examines technological innovations across the production and research processes of prepared food. Fourthly, from the consumer perspective, it examines the principal elements impacting customers’ purchasing and consumption patterns regarding prepared food. The current research has established a comprehensive framework for industrial safety assurances. Nonetheless, it exhibits notable inadequacies in viewpoints. As the final consumers of the products, research on the perceived risks of food safety in prepared food has consistently been a weak link, revealing a significant gap in the systematic analysis of the formation logic and key influencing factors of consumers’ perceived risk, necessitating further exploration. This study seeks to address a research gap by examining consumers perspective and identifying factors that influence their perceptions of risk associated with the safety of prepared food. This offers a theoretical basis for comprehending how these elements influence the perceived risk of prepared food.

## 3. Theoretical Foundation and Research Hypotheses

### 3.1. Perceived Risk Theory on Food Safety

The risk perception hypothesis was introduced by Bauer in the context of consumer behavior research [11]. The theoretical basis originates from the psychological concept of risk perception. This hypothesis challenges the conventional notion of total rationality in consumer behavior research, suggesting that customer decisions are impacted not just by factual information but also by subjective risk perception. Bauer characterized risk perception as comprising two fundamental dimensions: uncertainty (the potential for outcomes to diverge from expectations) and negative consequences (the likelihood of adverse effects), highlighting that risk perception is a subjective psychological construct influenced by individual experience, information gathering, and product attributes. In later research, Peter and Tarpey further delineated the concept of risk into two dimensions: perceived probability and perceived severity [12]. Finucane et al. identified the significant influence of emotional heuristics on risk-reward assessments, elucidating the psychological mechanism whereby positive emotions amplify gain perceptions while diminishing risk evaluations [13]. Antle highlighted the equilibrium between the scientific basis of risk assessment and the cost-effectiveness of regulation, emphasizing the faith in product qualities from a food safety standpoint [14]. These investigations together developed a multi-dimensional analytical framework for risk perception theory, offering a systematic theoretical foundation for comprehending consumers’ risk assessment behaviors in intricate decision-making contexts.

As research scenarios broadened, scholars discovered that the dimensions of perceived risk across various product categories change, rendering a singular “probability-severity” two-dimensional framework inadequate for comprehensively encompassing customers’ risk perception. Jacoby and Kaplan first divided perceived risk through empirical research into five specific dimensions: financial risk (economic losses due to quality problems after purchasing the product, such as wasted money due to spoiled prepared food that cannot be eaten), functional risk (the risk that the product fails to achieve the expected function, such as the taste, nutrition, and promotion of prepared food not matching after heating), physical risk (the risk that the product causes harm to the consumer’s health, which is the core dimension of food safety perceived risk for prepared food, such as excessive microorganisms and the abuse of additives leading to health problems), psychological risk (negative emotions caused by improper product purchase, such as anxiety about health problems after consuming prepared food), and social risk (the impact on one’s social image due to purchasing a certain product, such as a decline in social evaluation due to safety problems after recommending prepared food to others) [15].

Consequently, owing to the specificity of the food sector, researchers further refined the categorization of perceived risk dimensions. Mitchell identified in his research on food consumption behavior that physical risk and functional risk are the two primary categories of concern for consumers. In the prepared food sub-segment, the unique characteristics of the product, including transparency in processing, challenges in source traceability, and storage and transportation requirements, intensify consumers’ perception of physical risk [16]. Concurrently, they become increasingly aware of information asymmetry risk, which arises from their inability to acquire comprehensive information regarding the production, processing, and quality inspection of prepared food, leading to the perception of potential safety hazards. This enhancement to the dimension renders the perception risk theory more relevant to real-world contexts in food safety research for prepared food, offering a more accurate theoretical foundation for the subsequent examination of influencing factors.

The suitability of perception risk theory for researching food safety in prepared food stems from its capacity to effectively elucidate the relationship between the attributes of prepared items and customers’ risk awareness. Prepared food is classified as composite attribute products, encompassing both experiential products and trust products. Consumers must engage in consumption to assess taste, flavor, and other functional attributes. Conversely, food safety attributes—such as the freshness of raw materials, the absence of prohibited additives, and the hygiene of the production environment—cannot be evaluated through visual inspection or immediate experience; they depend on information supplied by producers or third-party certifications. The characteristic of information asymmetry is the fundamental cause of the emergence of perception risk [17]. From the standpoint of consumers’ cognitive reasoning, the consumption contexts of prepared meals predominantly pertain to familial daily dining and accessible snacks, encompassing vulnerable demographics such as youngsters and the elderly. Consumers possess an elevated anticipated standard for food safety. Upon recognizing potential hazards, their dual assessment of “likelihood–severity” will become more rigorous. For instance, when consumers observe the long shelf-life label on prepared meals, they may subjectively heighten their perception of the risk associated with excessive additives (physical risk), while concurrently experiencing psychological anxiety due to concerns for their health (psychological risk). This multi-faceted risk perception is layered, ultimately influencing their purchase choices. The perception risk theory, by systematically categorizing risk dimensions and analyzing influencing mechanisms, elucidates this cognitive process, enabling researchers to discern which factors (such as information transparency, brand trust, and consumption experience) significantly impact consumers’ risk perception levels. This, in turn, offers a theoretical foundation for devising strategies to mitigate perception risk regarding food safety in prepared food and bolster consumer confidence.

Current studies primarily focus on the single-agent trust mechanism, and consensus about the dynamic interaction effects of multi-agent collaborative trust remains elusive. Consumers’ risk perception may simultaneously be affected by three factors: nutritional balance, technical safety, and governance trust. This study aimed to develop a comprehensive research model examining the influence of customer risk perception, taking into account the qualities of prepared food goods and consumer trust. We incorporated a research hypothesis into the model, which delineates the following pathway: (1) The nutritional health characteristics of prepared food will influence consumers’ perceived food safety risk, resulting in a substantial negative effect; (2) The nutritional health characteristics of prepared food will impact risk perception via the mediating variable of technical safety; (3) The level of trust can modulate technical safety and perceived risk, with a significant regulatory effect. Consequently, we put up the subsequent hypothesis.

### 3.2. Research Hypotheses

#### 3.2.1. Nutritional Balance

Recent studies have analyzed the nutritional and health implications of prepared food from various perspectives. Research indicates that the preparation of prepared food can lead to nutrient loss or alteration. Thermal sterilization of prepared meat products effectively reduces foodborne pathogens. However, it may also lead to nutrient and flavor degradation. The preparation of prepared food leads to a notable reduction in vitamins and minerals. Additionally, prepared food contains high levels of fat and sodium to meet consumer preferences or extend shelf life. The extended intake of these prepared foods may increase the likelihood of obesity and cardiovascular diseases [18]. In contrast, other studies suggest that the nutritional value of prepared food is not wholly inferior to that of freshly prepared meals. Research demonstrates that there is no significant difference in the concentrations of essential nutrients, such as protein, carbohydrates, and fats, between frozen prepared meals and freshly prepared meals [19]. Additionally, optimizing the selection of components and processing methods can significantly reduce the nutritional degradation of prepared food. Fresh fruits and vegetables rich in vitamin C may be chosen to augment nutrients that are potentially lacking in prepared meals [20]. The perception of the nutritional quality of prepared food, including its nutritional value, mineral, vitamin, and fiber content, significantly influences consumers’ purchasing behavior. Consumers exhibit a greater inclination towards choosing prepared food products that utilize low-sodium formulations and natural ingredients. Moreover, dishes lacking preservatives may reduce customers’ risk perception, while providing credible health indicators can significantly decrease consumers’ awareness of food safety threats [21].

#### 3.2.2. Technical Safety

The technological and safety challenges related to prepared food affect all stages of production, processing, packaging, and cold chain logistics, representing significant issues that necessitate prompt resolution for the continued progress of the prepared food industry. Technical safety encompasses the quality and durability of prepared food, significantly impacting customer health and their trust in the products. The complexity of the prepared food supply chain makes it vulnerable to various food safety risks. In the raw material procurement phase, numerous firms, aiming to minimize costs, compromise on the quality and safety of raw materials by employing low-quality or substandard products, thereby introducing potential risks to food safety [22]. In the preparation of prepared food, insufficient screening of raw materials can lead to the presence of pathogenic microorganisms or residual pharmaceuticals, resulting in food safety issues [23,24]. The inadequacy of processing methods, such as non-standard pretreatment and irrational sterilization techniques, may lead to microbial residues or nutrient depletion [25]. The safety of packing materials should not be overlooked. Some packing materials may contain harmful compounds that can migrate into food during storage and transportation, posing a risk to consumer health [26,27]. Temperature variations in cold chain logistics are significantly important. Insufficient temperature control may lead to the degradation of prepared food, thereby jeopardizing both product quality and safety. Recent technological advancements have somewhat alleviated technical safety concerns in the prepared food sector [28]. These technologies extend the shelf life of prepared food while maintaining nutritional integrity and flavor quality. The implementation of blockchain technology enhances the transparency and traceability of the supply chain for prepared food. The implementation of the blockchain traceability system has enabled real-time visualization of supply chain data. Consumers can obtain detailed information about the entire prepared food process, including raw material procurement, processing, packaging, and shipping, by utilizing QR codes and other methods, thereby significantly reducing concerns related to food safety. This technology enhances consumer trust and enables quality control and market oversight for companies.

#### 3.2.3. Governance Trust

Consumer trust in government agencies is crucial in decision-making and is intricately linked to their risk perceptions. Trust-risk decision-making model posits that trust reduces risks through two main mechanisms: the perception of institutional guarantees and the accumulation of relational history, thereby aiding consumers in decision-making under uncertainty. Food safety hazards linked to prepared food occur at multiple stages. Inadequate addressing of food safety concerns regarding prepared food will compromise customers’ rights to information and choice, leading to diminished trust in these products [29]. Studies on consumer trust and risk perception concerning prepared food have established models based on the theory of planned behavior, demonstrating that consumers attitudes, subjective norms, and perceived behavioral control significantly affect their purchase intentions [30]. The relationship between consumers’ perceived trust and their perceived risk remains under-researched. In the context of prepared food consumption, increased trust can mitigate the negative impacts of risk perception and influence the relationship between technical safety and risk perception through the attribution bias mechanism [31,32].

**Figure 7:**
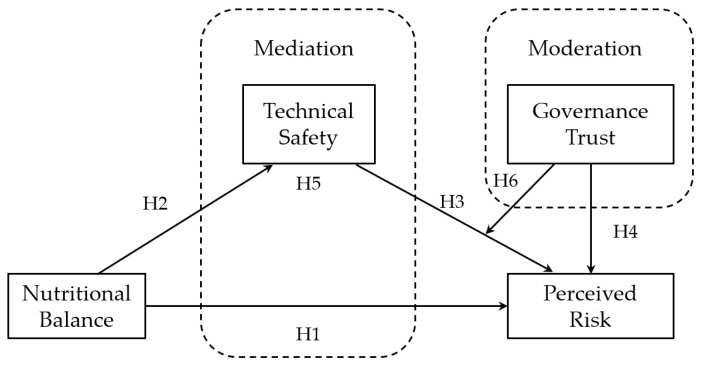
Research model on influencing factors of perceived risk of prepared food.

Based on the above analysis, this study proposes the following hypotheses. Figure 7 shows a research model diagram of factors affecting consumers’ perceived risk in prepared food market.

H1 The nutritional balance has a significant negative impact on perceived risk.

H2 The nutritional balance has a significant positive impact on technical safety.

H3 The technical safety has a significant negative impact on perceived risk.

H4 Governance trust has a significant negative impact on perceived risk.

H5 Technical safety has a mediating effect on nutritional balance and perceived risk.

H6 Governance trust has a significant moderating effect on technical safety and perceived risk.

## 4. Research Design

This study developed a research model to examine the factors influencing consumers’ perceived risk regarding food safety in prepared food. It utilized Zhengzhou, a significant food city in central China, as a case study for empirical analysis and testing of the key factors affecting consumers’ food safety risk associated with prepared food. The questionnaires were primarily distributed and collected via the Chinese online survey platform “Wenjuanxing”. This platform offers convenient usability, high efficiency in data collection, and secure, reliable data information. Constructing a complex causal relationship model facilitates a thorough exploration of the internal relationships and path effects among variables. This approach addresses unobserved variables and accounts for measurement errors, yielding more comprehensive and accurate analytical results.

This study advances the questionnaire design based on a literature review. Firstly, it systematically reviews the relevant literature in the fields of prepared food consumption, perceived risk, and food quality and safety, extracts the core variables, and constructs a research model in combination with relevant theories. Secondly, based on the core variables and the research model, it designs the initial measurement items for each dimension by referring to mature scales and considering the characteristics of prepared food. Then, it invites experts in the field to evaluate the initial questionnaire and collect revision suggestions. On this basis, a pre-survey is conducted to verify the reliability and validity of the questionnaire, and a formal questionnaire is formed. Finally, a large-scale questionnaire survey is carried out through a combination of online and offline methods to obtain data for empirical analysis.

This research focuses on consumers of prepared food in Zhengzhou for several reasons. Firstly, Zhengzhou serves as the capital of a province located in central China. This city is significant for its extensive food market and commercial sector. Secondly, Zhengzhou is classified as one of the 15 new first-tier cities in China, exhibiting a GDP of 1.36 trillion RMB, which positions it among the nation’s leaders. The population is 13 million, representing a significant market for prepared food consumption in China. The prepared food market in Zhengzhou has experienced rapid development. Urban residents exhibit greater openness to new concepts and possess a higher level of knowledge, rendering this location an optimal site for research. This research employed a questionnaire survey to assess the model’s validity. The study employed a five-point Likert scale to formulate questions aimed at measuring variables among consumers who have purchased prepared food. Throughout the investigation period, a total of 585 valid questionnaires were collected. Table 2 displays demographic data pertaining to consumers. The research protocol was reviewed and approved by the Ethics Committee of School of Management, Henan University of Technology, under approval number 20250328, approval date 28 March 2025. Informed consent was obtained from all participants prior to their participation in the study. The confidentiality and privacy of participants were ensured throughout the research process.

## 5. Model Analysis and Hypothesis Testing

### 5.1. Reliability Analysis and Validity Analysis

The reliability and validity of the scale data were assessed initially. The reliability of the data was evaluated through Cronbach’s Alpha. In reliability analysis, a Cronbach’s Alpha coefficient exceeding 0.7 signifies high reliability of the questionnaire, indicating that further analysis may be warranted. Table 3 presents the Cronbach’s Alpha coefficients for each dimension of the questionnaire in this study as 0.826, 0.810, 0.822, and 0.799, respectively. All values exceed 0.7, indicating a high level of reliability for the questionnaire [33]. It is also found that Cronbach’s Alpha and the combined reliability CR value are both greater than 0.7, and the average variance extraction AVE is both greater than 0.5. This indicates that the scale has good internal consistency and convergent validity. Table 4 shows the Discriminant validity test of the scale. It is found from the results that all the correlation indicators are smaller than the data with bold diagonals, indicating that the scale had good discriminant validity.

Additionally, factor analysis was utilized to evaluate the validity of the data structure of the scale. The KMO test and Bartlett’s sphericity test were performed to evaluate the suitability of the data for factor analysis. In validity analysis, a KMO score greater than 0.7 is generally regarded as indicative of the suitability of questionnaire data for factor analysis. Table 5 presents a KMO test result of 0.828, surpassing the 0.7 threshold, and a Bartlett sphericity test significance of 0.001, indicating substantial effectiveness at the 0.001 level, thereby confirming the data’s suitability for factor analysis [35].

Through further in-depth analysis, it can be concluded from Table 6 that the total variance that can be explained by the factors extracted from the questionnaire is 73.122%, indicating that the factor interpretation ability is good, and the extracted four factors can retain the original data information more completely. At the same time, the variance of extracting the first factor load without rotation is 36.408%, which is lower than 40%, indicating that there is no serious common method bias in the questionnaire.

The factor load presented in Table 7 indicates that all questions in the questionnaire align with the designated dimensions. The questionnaire demonstrates good validity, allowing the data collected to be utilized for subsequent analysis. The questionnaire demonstrates high reliability and validity, making it a reliable and effective tool for research and analysis.

The Pearson correlation coefficient was employed to examine the relationships among nutritional balance, technical safety, governance trust, and perceived risk. Table 8 indicates that the correlation significance between each variable and perceived risk was below 0.01, demonstrating a significant negative correlation.

### 5.2. Regression Analysis

The study examined the factors influencing perceived risk, with nutritional balance, technical safety, and governance trust as independent variables, and perceived risk as the dependent variable. Linear regression analysis was employed. Table 9 illustrates that the model’s R^2^ is 0.118, indicating a good fit. The independent variables account for 11.8% of the variance in the dependent variable. Additionally, the model’s significance is below 0.05, confirming that the model is adequately fitted and that at least one independent variable significantly influences the dependent variable. In conclusion, the model coefficients indicate that the Variance Inflation Factor (VIF) is significantly below 10, suggesting the absence of serious collinearity. The significance indicators of nutritional balance, technical safety, and governance trust are all below 0.05, indicating a substantial negative impact on perceived risk.

### 5.3. Mediation Effect Test

The Bootstrap method was employed to more accurately verify the mediation effect. The Bootstrap sampling was conducted 5000 times, with a confidence interval level of 95%. The mediation test results are presented in Table 10.

The path “nutrition balance → technical safety → perceived risk” exhibits an indirect effect of −0.109, with a confidence interval of [−0.151, −0.077], which does not include 0. This finding confirms the validity of the hypothesis regarding the intermediary effect in the relationship between nutrition balance, technical safety, and perceived risk.

### 5.4. Moderation Effect Test

**Figure 8:**
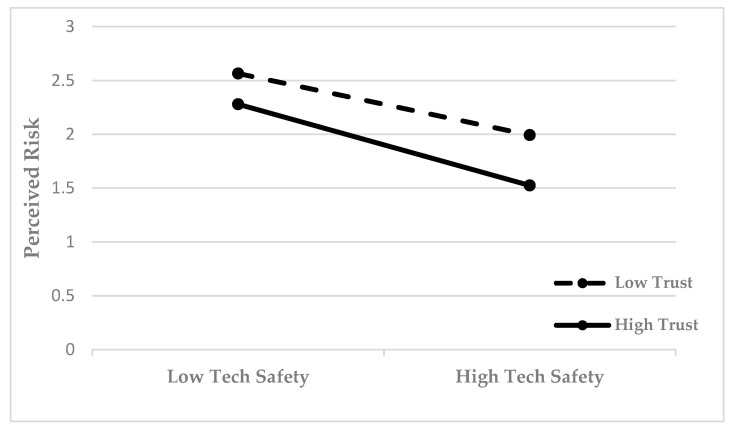
Changes in perceived risk along with technical safety and governance trust.

The process model 1 Bootstrap method was employed to conduct 5000 samples, with a 95% confidence interval utilized to assess the adjustment effect. Table 11 illustrates that, under conditions of low adjustment variables, the effect of technical safety on perceived risk was −0.171, with a *p*-value less than 0.05, signifying a statistically significant impact. The influence of technical safety on perceived risk, as indicated by medium adjustment variables, is −0.263, with a significance level of P less than 0.05. The influence of technical safety on perceived risk, in the context of high adjustment variables, is −0.354, with a significance level of P less than 0.05. Thus, a regulatory relationship exists between technical safety and perceived risk. Figure 8 shows how perceived risk changes with technical safety and governance trust. The horizontal coordinate indicates the degree of technical safety, from low technical safety to high technical safety. The ordinate represents perceived risk. Between low trust and high trust, perceived risk has a certain trend of change. In the low technical safety state, the perceived risk may be relatively high; In the high technical safety state, the perceived risk may be relatively low. Under different adjustment variables (low and high), technical safety has different degrees of influence on perceived risk, and the influence size is significant and gradually increasing.

The process model 14 Bootstrap method sampled 5000 instances, utilizing a 95% confidence interval to perform the moderated mediation effect test. Table 12 illustrates that the mediation effect size was −0.054, with a 95% confidence interval that did not include 0 under conditions of low moderating variables, indicating a significant effect. This indicates a mediating effect at this level. When the mediating variable is at a medium level, the mediating effect size is −0.097, with a 95% confidence interval that does not include 0, indicating a significant mediating effect. Conversely, when the mediating variable is at a high level, the mediating effect size is −0.139, also with a 95% confidence interval that does not include 0, confirming a significant mediating effect at this level. The moderated mediation effect test indicates that the 95% confidence interval does not include 0, thereby confirming the establishment of the moderated mediation effect. In a context of low technical safety, consumers assess the risk as relatively high. Consumers perceive a relatively low risk in the context of advanced security technology. Furthermore, increased consumer trust correlates with a more significant effect of technical safety on diminishing perceived risk.

Through data analysis, it is found that the nutritional balance of further affects consumers’ perceived risk by affecting technical safety. The moderating effect of consumer trust is significant. This indicates that consumers believe that the better the nutritional balance, technical safety or trustworthiness of ready-to-eat meals, the lower the risk level they perceive will be.

Figure 8 illustrates the relationship between perceived risk, technical safety, and governance trust. The horizontal axis denotes the level of technical safety, ranging from low to high technical safety, while the vertical axis reflects perceived risk. Perceived risk exhibits a distinct trend of variation between low trust and high trust. In a low technical safety state, the perceived risk tends to be higher, whereas in a high technical safety state, the perceived risk is generally lower. Technical safety exerts varying degrees of influence on perceived risk under different adjustment variables (low and high), with the magnitude of this influence being significant and progressively increasing.

## 6. Discussion

In the research framework for understanding the perceived risks of food safety in prepared foods, this study defines trust as a moderating variable. The difference in positioning between this study and other research, which treats trust as a direct variable, stems fundamentally from the different theoretical interpretations of the path of trust and risk perception. From the logical perspective of this study, the moderating effect of trust is manifested in the fact that it does not directly change the level of consumers risk perception, but rather exerts its influence by affecting the strength of the core transmission chain of “nutritional balance → technical safety → perceived risk”. For instance, when consumers have a high level of trust in the brand or category of prepared foods, the positive impact of nutritional balance on technical safety will be more significant, thereby strengthening the transmission effect of “nutritional superiority → technical safety credibility → low risk perception”. Conversely, if the trust level is low, even if the prepared foods demonstrate good nutritional balance attributes, consumers’ recognition of their technical safety will be weakened, ultimately resulting in a compressed reduction in the amplitude of risk perception. This characteristic of amplifying or weakening the core path effect is the typical mechanism of a moderating variable. While other studies consider trust as a direct variable, their theoretical basis focuses on the direct intervention of trust on risk perception: such studies believe that in the consumption scenario of food market, trust itself is the key reference for consumers to assess risks. When consumers trust the production standards, regulatory processes, or brand commitments of the prepared food enterprises, they will directly reduce their concerns about raw material contamination and processing violations and other safety risks, achieving a reduction in risk perception without the need for the mediation of other variables [36,37].

The differences in positioning further reflect the divergence in research perspectives: this study focuses more on analyzing how trust affects the relationships between other variables, paying attention to the contextual role in the interaction of variables, while studies that consider trust as a direct variable focus more on the independent impact of trust on the outcome variable, emphasizing the direct intervention of a single variable; the two are not in an opposing relationship, but reveal the value of trust in prepared food safety perception from different dimensions—the former highlights the correction effect of trust on the logic of consumer decision-making, while the latter emphasizes the fundamental value of trust as a risk buffer, jointly enriching the understanding of the trust mechanism in prepared food consumption.

Based on the analysis of the above results, this research further summarizes the results of the hypothesis test as shown in Table 13.

## 7. Conclusions and Implication

This study examined 585 consumers in a developing first-tier city in China and empirically explored the elements affecting consumers’ perceived risk associated with prepared food. Nutritional balance, technical safety, and governance trust adversely affected perceived risk of prepared food. The nutritional balance of prepared food directly influences perceived danger and indirectly affects it through the mediating variable of technical safety. Governance trust influences the effect and exerts a considerable impact. The study revealed that when customers consider the nutritional balance of prepared food as superior, they also regard the technical safety as elevated, thereby reducing their perceived risk. Moreover, an elevated level of governance trust correlates with a more significant influence of technical safety on diminishing the perceived risk associated with prepared food.

The results of this study may serve as a reference for the advancement of the prepared food industry and the regulation of food safety in governmental agencies. For prepared food enterprises, a visual production system is firstly suggested in the production process to improve product quality transparency. The firm might exhibit to consumers the production phases of prepared foods, encompassing raw material procurement, cleaning and processing, cooking and preparation, packing and sterilization, while also revealing the qualification details of food suppliers. This allows consumers to comprehend the production process of items and attain product traceability, consequently bolstering their belief in product quality. In the product design stage, the nutrition and labeling of the product can be optimized by precisely matching consumer demands. Diverse goods are tailored for distinct customer demographics, employing precise positioning to mitigate consumers’ perceived risks associated with nutritional imbalance. Simultaneously, product labeling is enhanced with comprehensive details regarding nutritional components and heating instructions to facilitate customer comprehension and mitigate risks associated with information asymmetry. In the marketing and after-sales stage, an efficient risk communication mechanism can be established to respond to consumers’ demands in a timely manner. Various communication platforms are created to disseminate scientific food safety information to consumers, mitigate cognitive dangers, and restore consumer trust. The government can enhance the standards of the prepared food industry by improving nutritional indicators, safety standards, and production norms, while also intensifying oversight of prepared food manufacturers to foster the healthy development of the market. To foster consumer trust, government agencies can mitigate perceived dangers and advance the sustainable development of the prepared food business by standardizing clear production processes and supplying comprehensive product information.

This investigation has yielded valuable insights; nonetheless, limitations persist. Owing to the constraints of the research parameters, we selected Zhengzhou, a representative new first-tier city in central China. Zhengzhou, while a representative sample city, may exhibit distinct risk perceptions compared to consumers in other first-tier cities. In the future, we will broaden the survey’s scope to encompass established first-tier cities like Beijing and Shanghai, alongside growing new first-tier cities such as Wuhan and Chengdu, enabling comparative research across many regions. By analyzing the variances in culture, purchasing power, and regulatory frameworks across various cities regarding customer perceived risks, we will further validate and refine the research model, thereby augmenting the universality and applicability of the research findings.

## Figures and Tables

**Table 1:** Citation analysis of journals and authors.

Ranking	Cited Journal	Counts	Ranking	Cited Authors	Counts
1	Plos One	176	1	Silvennoinen K	413
2	Food Chem	160	2	Adams J	275
3	Am J Clin Nutr	150	3	Zhang M	169
4	Food Control	134	4	Gogswell M E	51
5	J Agr Food Chem	131	5	Gooderham N J	40

**Table 2:** Descriptive statistical analysis of samples.

Demographic Variable	Item	Frequency	Percentage
Gender	Male	291	49.7%
	Female	294	50.3%
Age	≤30	153	26.2%
	31 to 40	209	35.7%
	41 to 50	122	20.9%
	>50	101	17.3%
Educational Background	High school and below	175	30.0%
	Undergraduate	304	52.0%
	Master and above	106	18.1%
Monthly household income	5000 yuan and below	231	39.5%
	5001–10,000 yuan	208	35.6%
	10,001 yuan and above	146	25.0%

**Table 3:** Factor loading, Cronbach’s Alpha, CR value and AVE value.

Dimension	Item	Factor Loading	CR	AVE	Cronbach’s Alpha	Reference
Nutritional balance	Prepared food can provide all the necessary nutrients for daily consumption.	0.801	0.8267	0.614	0.826	[34]
	Prepared food can be created with minimal use of preservative additives.	0.795				
	Prepared food serve as a means to minimize the use of salt and oil.	0.754				
Technical safety	The production process of prepared food may incorporate additives in accordance with regulatory standards.	0.780	0.8103	0.5874	0.810	[26]
	The production technology employed in the processing of prepared food is considered safe.	0.750				
	Prepared foods are considered safe during transportation and storage.	0.769				
Governance trust	The government can implement policies to promote the high-quality development of the prepared food market.	0.797	0.8225	0.6071	0.822	[30]
	The government departments possess the capacity to effectively oversee food safety within the prepared food market.	0.758				
	The government departments demonstrate a proactive commitment to overseeing food safety within the prepared food market.	0.782				
Perceived risk	I am highly concerned about the overall qualifications of food enterprises in the current prepared food market.	0.783	0.7991	0.5705	0.799	[29]
	I am apprehensive about the safety of the basic materials employed in the production of prepared food.	0.710				
	The news media’s exposure has prompted apprehension regarding the potential hazards associated with my consumption of prepared food.	0.771				

**Table 4:** Discriminant validity test.

	Discriminant Validity (Pearson Correlation)			
	Nutritional Balance	Technical Safety	Governance Trust	Perceived Risk
Nutritional balance	0.784			
Technical safety	0.537	0.766		
Governance trust	0.449	0.455	0.779	
Perceived risk	−0.289	−0.390	−0.274	0.755

**Table 5:** KMO and Bartlett sphericity tests.

KMO Sample Appropriateness Measure.		0.828
Bartlett sphericity test	Approximate chi-square	2749.507
	Degree of freedom	66
	significance	0.001

**Table 6:** Total variance interpretation.

	Initial Eigenvalue			Extract the Sum of Squared Loads			Rotating Load Sum of Squares		
	Total	Percent Variance	Cumulative %	Total	Percent Variance	Cumulative %	Total	Percent Variance	Cumulative %
1	4.369	36.408	36.408	4.369	36.408	36.408	2.223	18.523	18.523
2	1.766	14.717	51.125	1.766	14.717	51.125	2.222	18.517	37.040
3	1.432	11.930	63.055	1.432	11.930	63.055	2.166	18.050	55.090
4	1.208	10.067	73.122	1.208	10.067	73.122	2.164	18.032	73.122
5	0.477	3.975	77.097						
6	0.468	3.898	80.995						
7	0.440	3.664	84.659						
8	0.418	3.480	88.139						
9	0.386	3.214	91.353						
10	0.375	3.128	94.481						
11	0.337	2.810	97.292						
12	0.325	2.708	100.000						

**Table 7:** Component matrix after rotation.

Item	Ingredient			
	1	2	3	4
Nutritional balance 1	0.812			
Nutritional balance 2	0.836			
Nutritional balance 3	0.827			
Technical safety 1				0.827
Technical safety 2				0.794
Technical safety 3				0.807
Governance trust 1		0.858		
Governance trust 2		0.804		
Governance trust 3		0.827		
Perceived risk 1			0.823	
Perceived risk 2			0.825	
Perceived risk 3			0.837	

**Table 8:** Correlation coefficient matrix.

	Nutritional Balance	Technical Safety	Governance Trust	Perceived Risk
Nutritional balance	1			
Technical safety	0.439 **	1		
Governance trust	0.370 **	0.374 **	1	
Perceived risk	−0.234 **	−0.313 **	−0.223 **	1

Note: ** represent at level 0.01 (two-tailed), the correlation was significant.

**Table 9:** Results of linear regression analysis.

	Unnormalized Coefficient		Standardization Coefficient	t	Sig.	VIF
	B	Standard Error	Beta			
(Constant)	4.282	0.152		28.103	0.000	
Nutritional balance	−0.090	0.043	−0.094	−2.109	0.035	1.319
Technical safety	−0.230	0.044	−0.234	−5.209	0.000	1.324
Governance trust	−0.099	0.042	−0.101	−2.328	0.020	1.238
R^2^	0.118					
The adjusted R^2^	0.113					
F	25.831					
P	0.000					

Note: Dependent variable: perceived risk.

**Table 10:** Test of the mediation effect of Bootstrap.

Path	Effect Type	Effect Size	Standard Error	Bootstrap 95% CI		Relative Effect
				Lower Limit	Upper Limit	
Nutrition balance → Technical safety → Perceived risk	Total effect	−0.224	0.039	0.210	−0.234	
	Direct effect	−0.115	0.042	−0.108	−0.120	51.34%
	Indirect effect	−0.109	0.020	−0.151	−0.077	48.66%

**Table 11:** Simple slope analysis.

Regulating Variable	Effect	SE	t	P	LLCI	ULCI
Mean − SD	−0.171	0.058	−2.935	0.003	−0.286	−0.057
Mean	−0.263	0.041	−6.354	0.000	−0.344	−0.181
Mean + SD	−0.354	0.059	−6.052	0.000	−0.469	−0.239

**Table 12:** Moderated mediation tests.

Level	Effect	SE	LLCI	ULCI
Mean − SD	−0.054	0.025	−0.103	−0.004
Mean	−0.097	0.021	−0.137	−0.057
Mean + SD	−0.139	0.028	−0.196	−0.087
Moderated mediation tests	−0.039	0.015	−0.070	−0.010

Note: LLCI refers to the lower limit of the 95% range of estimates, ULCI refers to the upper limit of the 95% range of estimates.

**Table 13:** Hypothesis test result.

Research Hypothesis	Result
The nutritional balance has a significant negative impact on perceived risk.	H1 established
The nutritional balance has a significant positive impact on technical safety.	H2 established
The technical safety has a significant negative impact on perceived risk.	H3 established
Governance trust has a significant negative impact on perceived risk.	H4 established
Technical safety has a mediating effect on nutritional balance and perceived risk.	H5 established
Governance trust has a significant moderating effect on technical safety and perceived risk.	H6 established

## Data Availability

The original contributions presented in the study are included in the article, further data inquiries can be directed to the corresponding author.
